# Research progress on non-invasive testing of endometrial receptivity

**DOI:** 10.3389/frph.2026.1812188

**Published:** 2026-05-07

**Authors:** Lvhua Lin, Yanzhen Huang, Ruizhen Zhu

**Affiliations:** 1 The First School of Clinical Medicine, Guangdong Medical University, Zhanjiang, Guangdong, China; 2 The Affiliated Yangjiang Hospital, Guangdong Medical University (People’s Hospital of Yangjiang), Yangjiang, Guangdong, China

**Keywords:** endometrial fluid, endometrial receptivity, transcriptomics, proteomics, lipidomics

## Abstract

Endometrial receptivity plays a crucial role in the process of embryo implantation and is a key factor affecting the success rate of assisted reproductive technology. Traditional ultrasound assessment of endometrial receptivity has poor accuracy. The development of endometrial receptivity array (ERA) detection technology and RNA sequencing (RNA-seq)-based molecular testing for endometrial receptivity has enabled personalized embryo transfer (pET) for some patients based on test results. However, as an invasive testing technique, this method requires abandoning embryo transfer during the examination cycle. With the advancement of molecular diagnostics and omics technologies, a series of novel detection methods have emerged. Studies on the transcriptomics, proteomics, and lipidomics of endometrial fluid can, to some extent, also reflect endometrial receptivity. This article primarily reviews the latest research progress in non-invasive RNA-seq-based endometrial receptivity testing (nirsERT).

## Introduction

Endometrial receptivity refers to the ability of the endometrium to allow embryo positioning, adhesion, penetration, and implantation at a specific time, which is a critical factor determining whether embryos can successfully implant. Impaired endometrial receptivity (ER) represents one contributing factor to infertility. A clinical study suggests that 25.9% of patients with repeated implantation failure (RIF) experience unsuccessful embryo transfers due to a displaced window of implantation (1). To identify the optimal “window of implantation,” decades of research have attempted to evaluate endometrial receptivity using ultrasound parameters such as endometrial thickness, morphology, volume, blood flow, and peristaltic frequency. However, significant limitations persist in clinical practice. The mechanism of endometrial receptivity is a complex physiological process. While these ultrasound parameters may show statistical associations with pregnancy outcomes, a single indicator is far from sufficient to accurately assess endometrial receptivity.

In 2011, Diaz-Gimeno et al. (2) developed a molecular diagnostic tool based on gene expression profiling that enables the assessment of endometrial receptivity and facilitates personalized embryo transfer. This tool, termed the endometrial receptivity array (ERA), comprises 238 genes. A prospective, interventional, multicenter clinical trial demonstrated that among patients with repeated implantation failure (RIF) initially identified as being in a non-receptive (NR) state, a subsequent ERA test confirmed a genuine displacement of the window of implantation (3). Following personalized embryo transfer scheduled according to the ERA-determined date, these patients achieved pregnancy rates (PR) and implantation rates (IR) comparable to those of RIF patients who were initially identified as receptive (3). However, other studies have indicated that in women without RIF, ERA-guided pET showed no benefit (4). In women with RIF, low-quality evidence suggests that pET may improve clinical pregnancy rates (CPR) (4).

An RNA-seq-based endometrial receptivity test (rsERT) technology combined with the Random Forest (RF) algorithm dynamically analyzes the expression levels of related genes in endometrial tissue to identify an individualized window of implantation (WOI). The team's retrospective study (5) evaluated personalized embryo transfer (pET) based on rsERT and demonstrated that pET significantly improved pregnancy, implantation, and live birth rates compared with a transfer strategy based on the endometrial surface structural marker pinopode. Additionally, the multiple pregnancy rate in the pET group was significantly higher than in the conventional embryo transfer (ET) group. The study suggested that rsERT-guided pET has high clinical potential (6, 7).

Compared with ERA, although rsERT requires less tissue, it remains an invasive detection method that requires obtaining tissue samples through invasive endometrial biopsy. Patients cannot undergo embryo transfer in the same cycle as the test. Therefore, finding more effective, non-invasive methods to detect endometrial receptivity is a current research focus.

Uterine fluid (UF) is a mixture of endometrial glandular secretions, plasma transudate, and oviductal fluid. Its composition is dynamic and specific, adapting to meet the particular requirements of the embryo at different developmental stages, and it constitutes the liquid microenvironment for the blastocyst prior to and during implantation (8). Endometrial fluid contains cytokines, chemokines, proteases, antiproteases, and other substances that regulate processes related to blastocyst implantation. Exosomes/microvesicles released by the endometrium are also present in uterine fluid (9). These exosomes contain diverse proteins. Critically, the exosomal proteins are associated with fundamental processes of implantation: cellular adhesion, directional migration, invasive behavior, and dynamic remodeling of the extracellular matrix (10). Furthermore, UF is rich in glycoproteins, RNA, ions, lipids, and other components (11), and its composition is also influenced by immune cells. The composition of endometrial fluid changes dynamically with the menstrual cycle, and its specific patterns may reflect the state of endometrial receptivity, providing the possibility for developing non-invasive endometrial receptivity testing technologies based on endometrial fluid analysis. Studies have shown that fluid aspiration performed before embryo transfer does not adversely affect pregnancy rates in IVF cycles (12–14). Research on endometrial receptivity has benefited from advances in the “omics” field and the maturity of high-throughput tandem mass spectrometry technology. These technologies can identify thousands of molecules in minute samples, enabling quantitative detection of trace genes and proteins. Transcriptomics, proteomics, metabolomics, and lipidomics have made preliminary progress in this field, deepening our understanding of this complex physiological process.

## 1 UF collection methods

Currently, the primary methods for obtaining UF are lavage and direct aspiration. Under abdominal ultrasound guidance, an embryo transfer catheter connected to a 10 mL syringe is used to aspirate 5 to 50 microliters of endometrial fluid (15). Samples are extracted by gently pulling the syringe to create negative pressure. Withdraw the suction upon reaching the internal cervical os to prevent contamination by cervical mucus. During the procedure, special care should be taken to avoid touching the uterine fundus or causing cervical injury to minimize sample contamination by blood or endometrial tissue. In contrast, uterine lavage fluid is easier to obtain, typically by injecting 1–2 mL of physiological saline first. However, due to the unquantifiable loss of fluid through the fallopian tubes during lavage, it is difficult to determine the dilution level of molecular markers in the obtained specimen, making the measurement of extremely low-concentration markers more challenging (16). Due to the very small volume of aspirated fluid, floating cells cannot be removed before freezing, which may lead to the detection of some structural proteins (17). Two-dimensional differential gel electrophoresis (2D-DiGE) analysis of uterine lavage fluid is interfered with by the abundance of certain serum proteins, especially albumin and gamma globulin. If blood contamination occurs during sampling, hemoglobin can also cause interference. These proteins constitute approximately 90% of the total protein content in the sample, thereby masking lower-abundance proteins and making their detection and analysis difficult (18).

## 2 Transcriptomics in UF

A meta-analysis and validation study on genes associated with endometrial receptivity (19) revealed that 47 of the 57 meta-signature genes identified in the study overlapped with the core markers identified by the commercial endometrial receptivity diagnostic tool ERA. These overlapping genes are primarily involved in immune regulation, cell adhesion, exosome-mediated communication, signal transduction, and metabolism. Among them, the proteins of 28 endometrial receptivity-associated genes are present in exosomes, which can be secreted into the UF, suggesting that these genes/proteins in the UF may serve as biomarkers for non-invasive testing. Marzanati et al. (20) demonstrated that during the window of implantation, successful pregnancy was associated with heightened endometrial gene transcription and significantly elevated levels of four specific UF-EV-derived RNA transcripts (RPL10P9, LINC00621, MTND6P4, LINC00205) in the uterine fluid. Another study (21) compared the transcriptome of exosomes from endometrial aspirate fluid with the genes in the commercial ERA test, finding a high correlation. Specifically, on LH +7 vs. LH +2, 942 genes were upregulated and 1,305 were downregulated; in the pregnancy group vs. the non-pregnancy group, 97 genes were upregulated and 64 were downregulated (Table 1). These observations provide a basis for the development of non-invasive predictors of endometrial receptivity.

**Table 1:** The most significant core markers differentially expressed between the pregnancy group and the non-pregnancy group in this study.

Gene name	Expression trend (pregnancy vs. non-pregnancy)	Fold change (log2FC)	Main function and biological role
RPL10P9	Upregulated	> 1	May participate in protein synthesis regulation, providing energy and materials for embryo implantation
LINC00621	Upregulated	> 1	May be involved in epigenetic regulation of endometrial receptivity
MTND6P4	Upregulated	> 1	May be related to mitochondrial function and cellular energy metabolism
LINC00205	Upregulated	> 1	Involved in cell proliferation and differentiation regulation, may influence endometrial status
ZNF321P	Upregulated trend	1.12	May participate in transcriptional regulation, *p*-value close to significance (padj=0.051)
NAGS	Upregulated trend	1.26	Involved in urea cycle and amino acid metabolism, important for embryo development and endometrial microenvironment maintenance (padj=0.051)
BMP4	Upregulated trend	1.80	Key signaling molecule in embryonic development, involved in establishing endometrial receptivity (padj=0.058)

Sample information: The study included 82 IVF patients undergoing single vitrified-warmed blastocyst transfer (37 in the pregnancy group, 45 in the non-pregnancy group). Samples were collected during the secretory phase (implantation window, LH +7 days).

A study (22) aimed at identifying biomarkers from transcriptomic sequencing data of endometrial fluid delineated the expression profiles of 864 differentially expressed genes (DEGs) across three states of endometrial receptivity (ER): pre-receptive, receptive, and post-receptive phases. Among these, 468 genes overlapped with DEGs previously identified in rsERT studies (23), suggesting their common role in endometrial receptivity; conversely, the remaining 396 genes were unique to the uterine fluid. Integrated analysis further revealed that these shared DEGs were enriched in components such as extracellular vesicles, cytoplasm, nucleoplasm, and protein binding, providing robust evidence supporting the inference that uterine fluid RNA originates from endometrial cells and is secreted via vesicles. Notably, the 396 uterine fluid-specific genes showed significant enrichment in integrin-mediated signaling pathways and immune response processes, including leukocyte migration, inflammatory responses, and responses to lipopolysaccharide. This suggests that endometrial fluid, potentially through its unique adhesion and immunoregulatory functions, may play a distinct and critical role in the embryo implantation process.

Currently, there are few studies on UF transcriptomics, and they face a dual dilemma: insufficient RNA content in samples—nearly 1/3 of samples have total RNA <0.25 ng/*μ*L (22); and generally low technical reproducibility. This phenomenon can be attributed to discrepancies in sampling time points between studies, degradation by RNases, sample contamination (e.g., from blood, a small number of endometrial epithelial cells, or immune cells), and the diversity of RNA analysis techniques and marker gene screening methods. These factors collectively lead to sequencing difficulties, and different detection methods directly affect the reliability of results. Furthermore, the data analysis skills and database application capabilities of experimental personnel are also key variables determining research accuracy.

## 3 Application of proteomics in UF

Proteomics, which involves the systematic study of the complete set of proteins in a given biological system and their dynamic interactions, can provide more physiologically relevant information than transcriptomics. This is because multiple levels of regulatory mechanisms exist between the transcriptome and functional proteins, including post-transcriptional regulation, translational regulation, and post-translational modifications (24). UF is rich in proteins. Proteomic studies have confirmed that uterine fluid contains at least 600–1,500 different proteins (25, 26), with the specific number depending on the sampling and analytical methods used. The enrichment levels of 159 uterine fluid extracellular vesicle (UF-EV) proteins vary at different time points, indicating that the UF-EV proteome is cycle-dependent (27). A study obtained uterine fluid (UF) via uterine lavage from six women with normal fertility. Urinary luteinizing hormone (LH) testing determined the LH peak day (LH +0), and UF samples were collected on LH +1 to +3 and LH +7 to +9 days for proteomic analysis. The results showed that 367 proteins changed significantly during the transition from the early secretory phase to the mid-secretory phase (28). In this study, iterative testing based on the Random Forest (RF) algorithm was employed to perform a hierarchical analysis of 45 proteins that exhibited significant changes during the transition from the early secretory endometrium (ESE) to the mid-secretory endometrium (MSE), according to their expression abundance. The findings revealed that a protein panel consisting of PGR, NNMT, SLC26A2, and LCN2 achieved both specificity and sensitivity of 91.7% in distinguishing MSE from ESE (Table 2). However, only PGR, NNMT, and SLC26A2 were considered to play roles in endometrial (EM) receptivity, while LCN2 was only considered related to human EM function (28).

**Table 2:** Recent mass spectrometry-based analyses of endometrial fluid proteins related to receptivity.

Literature	Cycle type	Sample size	Sampling time	Key proteins	Function
Fitzgerald et al. (2018) (26)	Natural	19(fertile/infertile)	Secretory phase	ECM1, TGM2, IGHG4, CD44	Tissue regeneration, immunity
Kasvandik et al. (2020) (28)	Natural	47	Secretory/Proliferative phase	PGR, NNMT, SLC26A2, LCN2	Cell invasion, hormone metabolism

## 4 Application of lipidomics in UF

Endometrial fluid, particularly the microenvironment provided by glandular secretions, is crucial for embryo implantation (9). A study (29) analyzed uterine lavage fluid from cows on days 0, 3, 5, and 7 post-ovulation, identifying a total of 147 metabolites, including amino acids, benzoic acid, lipid molecules, carbohydrates, and vitamins. The concentrations of these metabolites were found to change significantly with the day post-ovulation, reaching peak levels on day 5 or 7, and these changes may be involved in regulating endometrial development prior to embryo implantation. Among the numerous metabolites in UF, lipids and their derivatives, particularly prostaglandins, are most closely associated with studies on endometrial receptivity. Prostaglandins (PGs), in particular, are perhaps the most intensively studied lipid molecules in this field. Furthermore, advances in mass spectrometry and nuclear magnetic resonance have enabled precise lipid detection. Research findings (30) indicate that prostaglandin E(2) and F(2*α*) are especially abundant during the window of implantation, where they may play a role in nourishing the blastocyst at the time of implantation and may also serve as important biomarkers for defining the receptive phase of the endometrium. Based on results from multiple animal model studies, the role of prostaglandins (PGs) in embryo implantation is widely recognized. Some studies suggest that reduced PGs synthesis in the human endometrium is a major cause of repeated implantation failure in patients undergoing IVF treatment (31). A study performed lipidomic analysis on uterine fluid samples collected during the natural menstrual cycle of healthy women. Nine specific lipids were identified, and the concentrations of prostaglandin E2 (PGE2) and prostaglandin F2*α* (PGF2*α*) in these samples were found to be significantly elevated during the window of implantation (WOI), particularly between days 19 and 21 (30). A comparative study found that patients with repeated implantation failure have defects in endometrial prostaglandin synthesis (31). The concentrations of PGE2 and PGF2*α* in human endometrial fluid are considered biomarkers for embryo implantation (32).

A study using matrix-assisted laser desorption/ionization time-of-flight mass spectrometry (MALDI-TOF) technology compared the lipidomic characteristics of endometrial fluid from patients with successful and failed frozen-thawed embryo transfer (FET) cycles. The study suggested that increased ratios of triglycerides and phospholipids could alter the endometrial window of implantation (33). Another study conducted a differential expression analysis of 294 lipids. Compared to patients with successful implantation, those with implantation failure exhibited significant alterations in eight lipids, comprising seven glycerophospholipids and one polyunsaturated fatty acid (Table 3) (34).

**Table 3:** Eight differential lipid metabolites in the endometrial fluid of patients with successful vs. failed embryo transfer.

Lipid metabolite	Metabolite class	Trend (failure vs. success)	Function
LPE(20:5)	Glycerophospholipid	Significantly decreased	As a lipid signaling molecule, its reduced level may affect endometrial inflammatory status and signaling, unfavorable for embryo implantation
PE(16:1e/20:5)	Glycerophospholipid	Significantly decreased	A membrane structural component; its reduction may alter membrane fluidity and receptor function of endometrial epithelial cells
PC(40:8)	Glycerophospholipid	Significantly increased	Its abnormal elevation may be a marker of lipid metabolism disorder in the non-implantation endometrium
PC(18:0/18:1)	Glycerophospholipid	Significantly decreased	As a major membrane phospholipid, its reduced level may affect membrane integrity required for endometrial receptivity
PC(18:0/18:2)	Glycerophospholipid	Significantly decreased	Similar to PC(18:0/18:1), its reduction is associated with implantation failure, possibly affecting membrane physical properties
LPE(22:5/0:0)	Glycerophospholipid	Significantly decreased	Similar to LPE(20:5), as a signaling molecule, its downregulation may reflect unfavorable changes in the implantation endometrial microenvironment
Docosapentaenoic acid (22:5n-6)	Omega-6 polyunsaturated fatty acid	Significantly increased	As a precursor for synthesizing signaling molecules like prostaglandins, its elevation may lead to local inflammation or dysregulated angiogenesis
LPE(18:2/0:0)	Glycerophospholipid	Significantly decreased	Similar to other LPEs, consistently reduced in non-implantation cycles, suggesting a synergistic role in embryo implantation

Sampling time: Secretory phase (specifically the implantation window, prior to embryo transfer). Cycle type: IVF cycles (all fresh embryo transfer cycles, all patients received standard antagonist protocols). Sample size: 29 patients total (15 in the successful transfer group, 14 in the failed transfer group).

Lipid molecules have a certain impact on endometrial receptivity. However, there is currently no consensus on the specific concentrations of lipid molecules required to effectively regulate endometrial receptivity. Furthermore, substantial evidence indicates that dietary supplementation with polyunsaturated fatty acids (PUFAs) influences biosynthetic pathways such as prostaglandin synthesis and steroidogenesis, which play diverse roles in regulating reproductive function (35). Further research is needed to establish lipid molecules as marker molecules for endometrial receptivity.

## 5 Conclusion

Endometrial fluid omics testing is a non-invasive diagnostic tool based on UF. Aspiration of UF before embryo transfer does not affect embryo implantation rates. However, current omics research results on UF lack reproducibility across different institutions, which may be related to factors such as inclusion criteria for study subjects, specimen acquisition methods, experimental methods, and data processing methods. Various substances in UF related to endometrial receptivity are not yet applicable in clinical practice and require further research and validation to obtain sufficient data support. As clinicians, we hope for a simple, rapid, and accurate non-invasive method for assessing endometrial receptivity during the same cycle as embryo transfer, as its sampling procedure does not compromise the success rate of subsequent embryo implantation.
